# AKH Signaling in *D. melanogaster* Alters Larval Development in a Nutrient-Dependent Manner That Influences Adult Metabolism

**DOI:** 10.3389/fphys.2021.619219

**Published:** 2021-02-23

**Authors:** Bryon N. Hughson, MaryJane Shimell, Michael B. O’Connor

**Affiliations:** ^1^Department of Ecology and Evolutionary Biology, University of Toronto, Toronto, ON, Canada; ^2^Department of Genetics, Cell Biology and Development, University of Minnesota, Minneapolis, MN, United States

**Keywords:** adipokinetic hormone, AKH, corpora cardiaca, prothoracic gland, metabolism, development, dg2, PKG

## Abstract

Metabolism, growth, and development are intrinsically linked, and their coordination is dependent upon inter-organ communication mediated by anabolic, catabolic, and steroid hormones. In *Drosophila melanogaster*, the corpora cardiaca (CC) influences metabolic homeostasis through adipokinetic hormone (AKH) signaling. AKH has glucagon-like properties and is evolutionarily conserved in mammals as the gonadotropin-releasing hormone, but its role in insect development is unknown. Here we report that AKH signaling alters larval development in a nutrient stress-dependent manner. This activity is regulated by the locus *dg2*, which encodes a cGMP-dependent protein kinase (PKG). CC-specific downregulation of *dg2* expression delayed the developmental transition from larval to pupal life, and altered adult metabolism and behavior. These developmental effects were AKH-dependent, and were observed only in flies that experienced low nutrient stress during larval development. Calcium-mediated vesicle exocytosis regulates ecdysteroid secretion from the prothoracic gland (PG), and we found that AKH signaling increased cytosolic free calcium levels in the PG. We identified a novel pathway through which PKG acts in the CC to communicate metabolic information to the PG via AKH signaling. AKH signaling provides a means whereby larval nutrient stress can alter developmental trajectories into adulthood.

## Introduction

Fundamental to all forms of life is the ability to maintain constancy of the internal milieu, or homeostasis, around precisely defined physiological setpoints (Bernard, 1854; Cannon, 1929). In *Drosophila melanogaster*, the stress-responsive neuroendocrine corpora cardiaca (CC) is ideally suited to this task (Vogt, 1946). The CC is the sole site of biosynthesis and secretion of the adipokinetic hormone (AKH), a glucagon-like peptide that mobilizes carbohydrate and lipid energy stores in response to starvation (Kim and Rulifson, 2004; Lee and Park, 2004; Isabel et al., 2005). The CC is functionally homologous to the mammalian pancreatic α cells. Perturbations in haemolymph glucose titers are detected by the CC through changes in the activity of evolutionarily conserved ATP-sensitive potassium (K_ATP_) channels (Kim and Rulifson, 2004). In murine pancreatic α cells, a cGMP-dependent protein kinase (PKG, encoded by *cGK1* in mice) negatively regulates glucagon secretion (Leiss et al., 2011). *cGK1* is evolutionarily conserved in *D. melanogaster* (*dg2*) and humans (*PRKG1*) (Kalderon and Rubin, 1989; Hofmann et al., 2006). From the murine literature, we hypothesized that *dg2* acts in the CC to negatively regulate AKH secretion.

*dg2* was previously renamed *foraging* (Osborne et al., 1997). *for* was reported to encode naturally occurring allelic variants (rover, *for*^*R*^, and sitter, *for*^*s*^) that underlie a behavioral polymorphism (Sokolowski, 1980; de Belle et al., 1989). Precise excision of the P element 189Y altered sitter behavior morphs into rovers, and this was reported to identify a new *for*^*s*^ allele (i.e., *for*^189*Y*^) (Osborne et al., 1997). Because 189Y was thought to be excised from the *dg2* locus, Osborne et al. (1997) reported that *dg2* is the *for* gene. However, 189Y was excised from the *lilliputian* (*lilli*) locus, which encodes a transcription factor (Flybase Id FBal0193987, FB2020_06; FlyBase Id FBgn0000721, FB2020_06; FlyBase Id FBti0007080, FB2020_06). Osborne et al. (1997) in fact identified *lilli* as the *for* gene, and their work remains the only molecular identification of *for* (Sokolowski, 2006; Wang et al., 2008). Consequently, it must be stated clearly that the present study examined the metabolic consequences of manipulating the PKG-encoding gene *dg2*.

A role for AKH in early development and sexual maturation was hypothesized but never identified (Owusu-Ansah and Perrimon, 2014; Gàlikovà et al., 2015). The possibility of such a role is suggested by the conservation of specific amino acids between AKH and the mammalian gonadotropin-releasing hormone (GnRH; Lindemans et al., 2009; Zandawala et al., 2017). GnRH acts in the hypothalamus-pituitary-gonadal (HPG) axis to signal the onset of puberty by stimulating increased steroid hormone biosynthesis and secretion. Support for the possibility that GnRH-like function may be conserved in AKH lies in the orthology between its receptor, AKHR, and the gonadotropin-releasing hormone receptor (GnRHR; Staubli et al., 2002). Phylogenetic analyses identified orthology between AKH and GnRH, and demonstrated that these peptides were ligands for orthologous receptors, AKHR and GnRHR (Jékely, 2013; Mirabeau and Joly, 2013).

The developmental transition between sexually immature larvae and mature adults in *D. melanogaster* also depends upon the precisely timed activity of steroid hormones (ecdysteroids) (Mirth and Riddiford, 2007). Ecdysteroid secretion at the end of the third larval instar permits pupariation and sexual maturation. Ecdysteroids are actively secreted from the prothoracic gland (PG) via calcium-mediated vesicle exocytosis (Yamanaka et al., 2015). The PG is an endocrine tissue adjacent to the CC. CC axons containing AKH project into the PG but their biological significance has never been identified (Kim and Rulifson, 2004). The possibility that these projections secrete AKH to target the PG is intriguing in light of the AKHR/GnRHR orthology. If AKH functions in a manner analogous to GnRH, then the steroid-producing PG is a logical target for its activity. The role of AKH as a nutrient stress signaling molecule makes it ideally suited for integrating metabolic information with developmental processes.

Here we report that reduced expression of *dg2* in the CC altered larval development, and that AKH signaling regulated these effects in a nutrient-dependent manner. Larvae reared in a low nutrient environment were developmentally delayed and showed increased lethality prior to pupariation. Flies that survived to adulthood were more starvation resistant, and had a greater proportion of whole body lipid content to body size than controls. AKH peptide abundance was reduced in the larval CC, and we identified the potential for paracrine secretion from the CC into the PG. We used *in vivo* calcium imaging techniques to show that AKH signaling in the PG increased cytosolic free calcium levels. These data identified an AKH-mediated signaling pathway that targeted the PG, either indirectly or directly via a novel CC-PG paracrine signaling pathway. This pathway might optimize the timing of developmental transitions in response to nutritional stress.

## Materials and Methods

### Fly Stocks and Husbandry

Stocks used in the present study were: a *dg2* genetic background stock bore isogenic first, second, and third chromosomes (Allen et al., 2017), *akh*Gal4 (BDSC 25684), UAS-*Dcr2* (BDSC 24650), UAS-*dg2*-RNAi (Dason et al., 2019), UAS-*dg2*-RNAi (VDRC GD38320), UAS-*gfp*-RNAi^142^ (BDSC 44415), UAS-*dTrpA1.k* (BDSC 26263), UAS-*CD4*:*td= .Tom* (BDSC 35841), 10XUAS-IVS-*mCD8:gfp* (BDSC 32185), UAS-*nSyb-e.gfp* (BDSC 6922), UAS-*ANF-EMD* (BDSC 7001), UAS-*GCaMP6m* (Chen et al., 2013), *spok*-Gal4 (Shimell et al., 2018), UAS-*AkhR*-RNAi (KK109300 and BDSC 29577). In order to control for genetic background, transgenes were backcrossed for nine generations into chromosome 2, and six to nine generations into chromosome 3 of the *dg2* sitter stock. VDRC KK109300 and TRiP BDSC 29577 were not backcrossed. In all experiments, homozygous transgenic parental stocks were crossed to produce F1 that were heterozygous for the transgenes, and F1 heterozygous controls were generated from transgenic stocks crossed with *dg2* genetic background stock.

AKH^A^, AKH^AP^ – loss of function mutants of AKH and AKH precursor-related peptide (APRP) – were generous gifts from Dr. R.P. Kühnlein (Gàlikovà et al., 2015). Third chromosome transgenes that were backcrossed into isogenic *dg2 background* for six to nine generations were backcrossed for two generations into AKH mutant third chromosomes. All AKH and AKHR mutant F1 used in experiments were homozygous for the mutant locus. AKHR^1^ was a generous gift from Dr. S. Grönke (Grönke et al., 2007). The AKHR1 second chromosome was not backcrossed into *dg2* genetic background. All AKH and AKHR mutant F1 used in experiments were homozygous for the mutant locus.

Stocks were reared at 25°C and 60% RH. Transfer of adults between 18 and 30°C for the temporal and temperature manipulations took place within 1 h of eclosion. Rearing at 18 and 30°C was in 60% RH. A 12:12 L:D cycle was used in all rearing and experimental conditions.

Normal nutrient food consisted of 100 g sucrose, 50 g yeast, 13 g agar, 8 g potassium sodium tartrate tetrahydrate, 1 g potassium phosphate monobasic, 0.5 g sodium chloride, 0.5 g calcium chloride, 0.5 g magnesium chloride hexahydrate, 0.5 g iron (III) sulfate hydrate, all dissolved in 1L of water. Five milliliter of propionic acid was added after boiling. Food was poured into standard fly vials, 10 mL of food per vial (Diamed, Cat #GEN32-120) at <40°C. Low nutrient food had 25% of the sucrose (25 g) and yeast (12.5 g) of normal nutrient food. Flies used in experiments were reared in a reduced agar (i.e., 9 g/L from 13 g/L) medium in order to reduce inter-vial variation in the ability of larvae to chew and ingest food.

Grape plates contained 2.0 g agar, 45 mL Welch’s grape juice, 50 mL water with 2.5 mL acetic acid and 2.5 mL 95% ethanol added at <60°C. The grape juice-agar mixture was poured into lids taken from 35 mm × 10 mm petri dishes (Falcon, #351008) and allowed to set overnight. For egg-laying, parents were added to fly bottles (Fischer Scientific, #AS355) that were subsequently capped with the grape plate petri dish lid. A drop of liquid yeast paste was placed on the grape plate before capping. These bottles had a circular opening in their side plugged with a sponge to allow for air flow. Parents laid eggs for 2–3 h. Larvae were picked within 30 min of egg hatching.

### Starvation Resistance Assay

As described in Hughson et al. (2018), starvation vials were made using standard fly vials (Diamed, Cat #GEN32-120) containing 10 mL of 1% agar and a cotton ball soaked with water. Virgin female flies were reared on standard food at 25°C in a 12:12-h light/dark cycle. Groups of 10 flies (*n* = 10; 7 ± 1 day old) were aspirated into each vial and the number of dead flies was counted every 6–8h. Starvation resistance was performed under specified rearing temperatures, light cycle and relative humidity.

### Proboscis Extension Response (PER) Assay

As described in Hughson et al. (2018), proboscis extension response (PER) assays were modified from Shiraiwa and Carlson (2007). Virgin female flies (7 ± 1 day old) were aspirated into 100 mL pipette tips so that only the head and one foreleg extended from the opening. Foreleg tarsi were stimulated by contact (<1 s) with Kim wipe tissue “threads” soaked with either water or various sucrose solutions (0.1, 0.3, 1, 3, 10, or 30%, all w/v). Sucrose treatments were presented in randomized order, and water was presented before and after each sucrose stimulus. A positive result was recorded for every full extension of the proboscis. Flies that responded positively to water were eliminated from analysis, as they were no longer responding exclusively to feeding cues. The number of positive responses for each fly was summed to yield an individual sucrose response (SR) score. The mean SR score was then calculated for each strain-trea tment group. Flies were scored separately to yield an individual and strain-averaged SR score. Flies were treated as described above for fed, 24 and 48 h FD groups. All experiments were conducted between 1,300 and 1,700 h.

### Triacylglyceride Quantification

Triacylglyceride (TAG) measurements were performed as described in Hughson et al. (2018). Feeding treatments were conducted as described above. Virgin females (*n* = 8; 7 ± 1 day old) were homogenized in 200 μL of 1× PBT with 0.5% TritonX (0.5% PBT-X). Another 800 μL of 0.5% PBT-X were added, the solution was vortexed, then heat inactivated at 70°C for 5 min. Samples were centrifuged at 5,000 rpm at 4°C for 1 min, the supernatant was removed and centrifuged again at 13,000 rpm for 3 min. Supernatant was removed and stored at −20°C for later use, or immediately pipetted into a Corning Falcon 96-well cell culture plate (VWR 351172): 50 μL of sample were added to each well, and three technical replicates were prepared for each sample. A blank reading of the plate was made at 562 nm using a BioTek Synergy HT spectrophotometer and Gen5 analytical software. Infinity TAG reagent (Thermo Scientific 796704) was preheated to 37°C and 200 μL were added to each well. The plate was incubated at 37°C for 15 min and read at 562 nm. Total TAG was determined using a standard curve made using a TAG standard (Trace DMA TR2291-030).

### Pearce Bicinchoninic Acid (BCA) Assay

As described in Hughson et al. (2018), protein was quantified using the Pierce bicinchoninic acid (BCA) protein assay (Thermo Fisher Scientific 23225). Supernatants (50 μL, prepared as per the TAG assay) were loaded in triplicate into a Corning Falcon 96-well cell culture plate (VWR 351172). The plate was read at 562 nm for a blank reading. BCA solution (150 μL) was added to each well. The plate was incubated at 37°C for 30 min and read at 562 nm. The blank reading was subtracted from the final reading and total protein content was determined using a standard curve made from a protein standard (BCA kit).

### Wing Measurements and Hair Counts

This method was adapted from Delcour and Lints (1967). The right wings of 7 ± 2 day old female adults were clipped and taped dorsal side up onto a slide. Relative wing size was measured by calculating the surface area within a polygonal region defined by the second costal break, the intersections of the lateral wing veins with the wing margin, and base of the aola. Images of wings were taken using a Zeiss epifluorescence microscope: wing area was quantified using FIJI and scaled using a micrometer slide; hair counts were made by hand from within a 100 μm × 100 μm area equidistant between the third and fourth lateral veins, and anterior to the posterior cross vein.

### Larval Development Assay

Flies (*n* = 100 females, *n* = 50 males; 3–6 days old) were placed on grape plates and allowed to lay eggs for 2–6 h. About *n* = 30 newly hatched (<0.75 h after egg hatching, AEH) L1 were seeded per vial. Numbers of wandering, pupariating and eclosing larvae/flies in each vial were counted every 1 h.

### Confocal Microscopy

A Leica TCS SP5 confocal laser-scanning microscope available through the University of Toronto Cell Systems Biology Imaging Facility was used for fluorescence imaging of all *ex vivo* immunohistochemistry and Nile red fat body staining, as well as *in vivo* calcium imaging.

### Immunohistochemistry

Freshly dissected tissues were fixed in 4% paraformaldehyde on ice for 25 min, and at room temperature (RT) for 30 min with rocking. Tissues were washed 2 × 5 min in 0.5% PBT-X (phosphate-buffered saline, Triton-X); all washes used 0.5% PBT-X with rocking at RT. Tissues were washed 4 × 30 min, blocked (14 μL 5% bovine serum albumin, 35 μL normal goat serum, in 651 μL 0.5% PBT-X) for 2h at RT, and incubated with rocking overnight (∼15 h) at 4°C in primary antibody solution. Tissues were washed 2 × 5 min, washed 4 × 30 min, incubated for 2h in secondary antibody solution at RT (culture plate was wrapped in tinfoil), washed 4 × 30 min, and rinsed 2 × 5 min in 1× PBS. Tissues were mounted on glass slides (VWR CA48323-185) with Vectashield-DAPI mounting medium (Vector Laboratories H-1200). Stored at 4°C, tissues could be imaged for up to 3 months. Primary antibodies were made in blocking solution at concentrations of 1:500 for mouseαGFP (mouse anti-GFP; Life Technologies A-11120), rabbitαGFP (Life Technologies A6455s), chickenαGFP (Life Technologies A10262), mouseαRFP (Thermo Scientific MA5-15257), and 1:600 for rabbitαAKH. rαAKH was a generous gift from Dr. Jae Park. Secondary antibodies were made in blocking solution at concentrations of 1:500: goatαrabbit Alexa Fluor 488 (Life Technologies 11008), goatαrabbit Alexa Fluor 568 (Molecular Probes A11011), goatαrabbit Alexa Fluor 633 (Invitrogen A21071), goatαmouse Alexa Fluor 488 (Life Technologies A11005), and goatαchicken Alexa Fluor 488 (Life Technologies A11039).

### Nile Red Fat Body Staining

Fat body staining was based upon Grönke et al. (2005). Briefly, freshly dissected fat body tissues were dissected and transferred to droplets of mounting medium on glass slides coated with 10% poly-L lysine. Mounting medium contained 50% glycerol and 0.5% PBT-X and 1:55,000 Nile red (Sigma, N3013-100MG) (stock solution of 10% Nile red in DMSO). Tissues were analyzed 6–12 h after mounting using a Leica SP5 confocal microscope.

### Calcium Imaging

Central nervous system-ring gland tissues (CNS-RGs) were dissected from the cuticle in Schneider’s insect medium (Sigma S0146) and transferred directly to imaging dishes containing a poly-L lysine-treated glass coverslip in 1,500 μL of haemolymph-like solution 6 (HL-6) with 4 mM calcium chloride; high trehalose concentration in HL-6 prolonged tissue survival, and reduced spontaneous calcium activity and AKH secretion (Macleod et al., 2002). Imaging dishes were constructed from 3.5 mm diameter culture dishes and thin (∼3 mm) sections cut from 2.5 mm diameter vials. The 2.5 mm vial sections were glued inside the 3.5 mm culture dishes to create the well in which the cover slip-tissue mounts were placed and imaged. Imaging was performed on a Leica TCS SP5 confocal laser-scanning microscope with a 63× water dipping objective (0.9 NA). Tissues were positioned on the coverslip and the objective focused on the PG. HL-6 (750 μL) was pipetted from the dish before the experiment started. At *t* = 5 min, 750 μL of HL-6 containing AKH peptide (Sigma Aqua peptide DAKH) was slowly pipetted into the dish over 1 min to a final concentration of 1 μM. Tetrodotoxin citrate (TTX, 100 nM; Abcam ab120055) was added with the HL-6/AKH solution in TTX experiments. CNS-RGs were scanned at 488 nm excitation at a speed of 1024 × 1024 every 5 s in 4 × 15 min blocks to permit refocusing as necessary. Total number of micro and macrospikes in each block was counted visually (Yamanaka et al., 2015).

### Statistics

**FIGURE 1:**
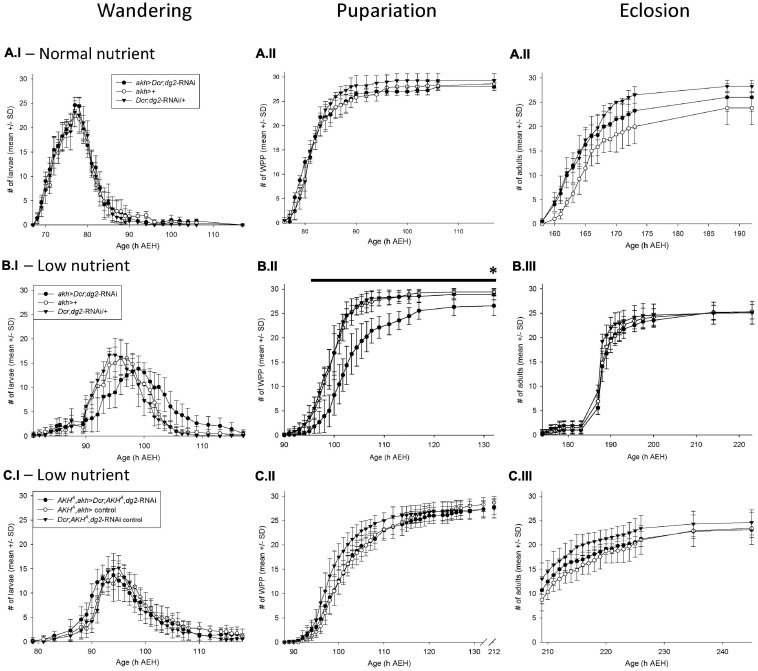
*akh* > *Dcr;dg2-RNA\* interacted with low nutrient stress to prolong development via AKH signaling Developmental timing of wandering, pupariation, and eclosion. The number of individual wandering larvae **(I panels)**, white prepuparia (WPP) **(II panels)** and adults eclosed **(III panels)** is recorded on the *y*-axis; the age of the flies (hours AEH) is on the *x*- axis. Each vial contained *n* = 30 newly hatched larvae att = 0h. Data are reported as mean ± standard deviation (SD). **(A.I–III)** In 100% standard nutrient rearing conditions, *akh* > *Dcr;dg2-RNA\* did not significantly affect wandering, pupariation or eclosion. *n* = 10 vials/genotype-treatment. **(B.I–III)** In 25% low nutrient rearing conditions, *akh* > *Dcr;dg2-RNA\* prolonged larval wandering, delayed pupariation, decreased survival to pupariation, and proportionately increased survival to eclosion. *n* = 10 vials/genotype-treatment. *Indicates significantly decreased survival in *akh* > *Dcr;dg2-RNA\* at 132h AEH (*p* < 0.05), black line indicates significant differences between *akh* > *Dcr;dg2-* RNAi and controls from 96h to 132h AEH (*p* < 0.05). **(C.I–III)** In 25% low nutrient rearing conditions, the effect of *akh* > *Dcr;dg2-RNA\* in a wild type AKH^+^ background was lost in the AKH^A^ single mutant background. *n* = 8 vials/genotype-treatment. See section “Materials and Methods” for details of statistical testing.

**FIGURE 2:**
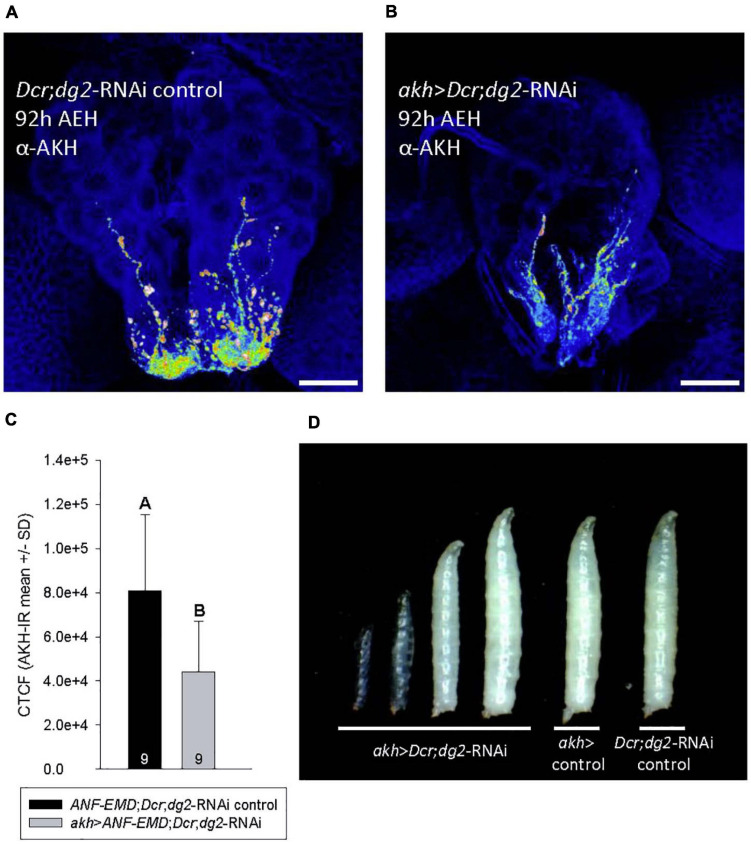
*akh* > *Dcr,dg2-RNM* altered AKH abundance and caused developmental arrest in low nutrient conditions. **(A–C)** Heat mapping projections from a-AKH revealed AKH-IR in CC cell bodies and axons in control 92h AEH L3 **(A)**, but AKH- IR was significantly reduced (*p* = 0.010) in *akh* > *Dcr;dg2-RNA\* larvae **(B,C)**. Corrected total cell fluorescence (CTCF) of AKH-IR was shown in panel **(C)**. *n* = 9 samples/genotype. Scale bar in panels **(A,B)** 20 μm. **(D)** Temporally age-matched larvae (92 h AEH) displayed developmental arrest at LI and L2 in *akh* > *Dcr;dg2-RNA\* larvae and a transparent “glassy” phenotype. See section “Materials and Methods” for details of statistical testing.

SigmaPlot Ver.12.5 statistical software was used for all statistical analyses. Larval development assay (LDA; Figures 1, 4E and Supplementary Figure 1) two-way ANOVA (multiple pairwise comparisons, Bonferroni). Total number of pupae (Figure 1B.II) were analyzed with two-way ANOVA (multiple pairwise comparison, Dunn’s). Immunoreactivity (Figure 2C) was analyzed with *t*-test (Mann–Whitney Rank Sum). Ecdysteroid titers (Supplementary Figures 4A,B) were analyzed with two-way ANOVA (multiple pairwise comparison, Holm-Sidak). Calcium spikes (Figure 4D and Supplementary Figure 4C) were quantified using FIJI^1^ and analyzed using two-way ANOVA (all pairwise comparison, Holm-Sidak). Starvation resistance assay (SRA; Figures 5A–C and Supplementary Figures 5A–E) were analyzed with Kaplan–Meyer survival analysis (multiple pairwise comparisons, Holm-Sidak). PER (Figure 5D), lipid content (Figure 5G), protein content (Supplementary Figure 5F), and wing measurements (Figures 5F,H) were analyzed with one-way ANOVA (Holm-Sidak).

**FIGURE 3:**
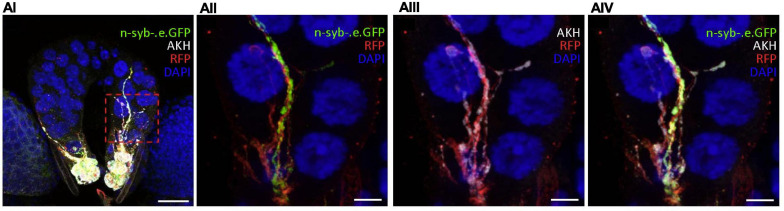
AKH might be secreted from the CC directly into the PG through a novel paracrine mechanism. **(AI–IV)** In the ring gland of 92h AEH *akh* > *td.Tom;nSyb.eGFP* larvae, GFP-tagged neuronal synaptobrevin and AKH-IR colocalize within CC axonal projections to the PG. Region of interest (red dashed box) in panel **(AI)** is magnified in panels **(AII–IV)**. Split GFP-IR [**(AII)** – green] and AKH-IR [**(AIII)** – white] channels are merged in panel **(AIV)**. DAPI (blue) and RFP intensity (red) were modified in order to better identify neuronal projections. Scale bars: **(AI)** 20 μm; **(AII–IV)** 5 μm.

**FIGURE 4:**
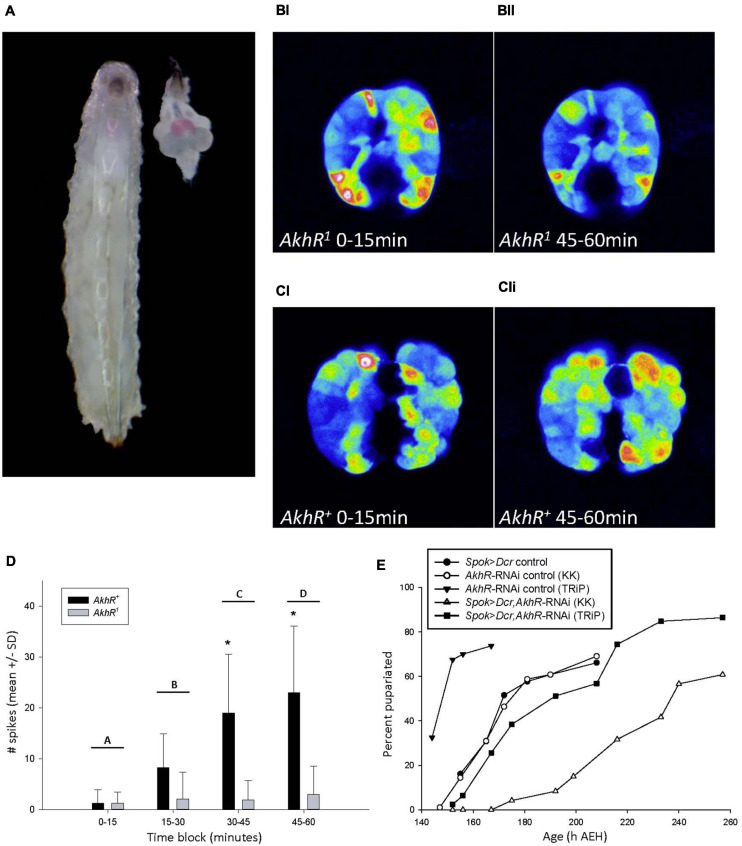
AKH signaling in the PG increased intracellular cytosolic free calcium levels. **(A)** 90hAEH L3 and a dissected CNS-RG positioned to match its anatomical location in the larva. The PG was stained pink by a chemical reaction caused by *spok-GAL4* driving the co-expression of *UAS-GCaMP6m* and *UAS-CD4-td. Tom*. **(B,C)** Maximum intensity projections of PG intracellular calcium activity recorded *in vivo* from 88 to 90 h AEH larvae. Maximum intensity projections of PG calcium spike activity representative of *AkhR*^1^ **(BI,II)** and *AkhR*^+^ **(CI,II)** *spok* > *GCaMP6m* larvae during the 0–15 min **(BI,CI)** and 45–60 min **(BII,CII)** blocks. Representative videos of changes in calcium spiking can be found in online supplementary information. **(D)** Calcium spike activity did not differ between *AkhR*^+^ vs. *AkhR*^1^ during the 0–15 or 15–30 min block, but increased significantly in *AkhR*^+^ in each subsequent block. *p < 0.001 denotes statistically significant differences between genotypes within a time block; letters A vs. C and D (*p* < 0.001), B vs. C (*p* = 0.006), B vs. D (*p* < 0.001), A vs. B (*p* = 0.079), and C vs. D (0.237) denote statistically significant differences between time blocks in *AkhR*^+^ (*p* < 0.05). **(E)** Reduced *AkhR* expression with KK (VDRC KK109300) in the PG caused significant developmental delay to pupariation in low nutrient food (*p* < 0.001). Reduced *AkhR* expression with TRiP (BDSC 29577) did not cause significant developmental delay (*p* = 1.0). See section “Materials and Methods” for details of statistical testing.

**FIGURE 5:**
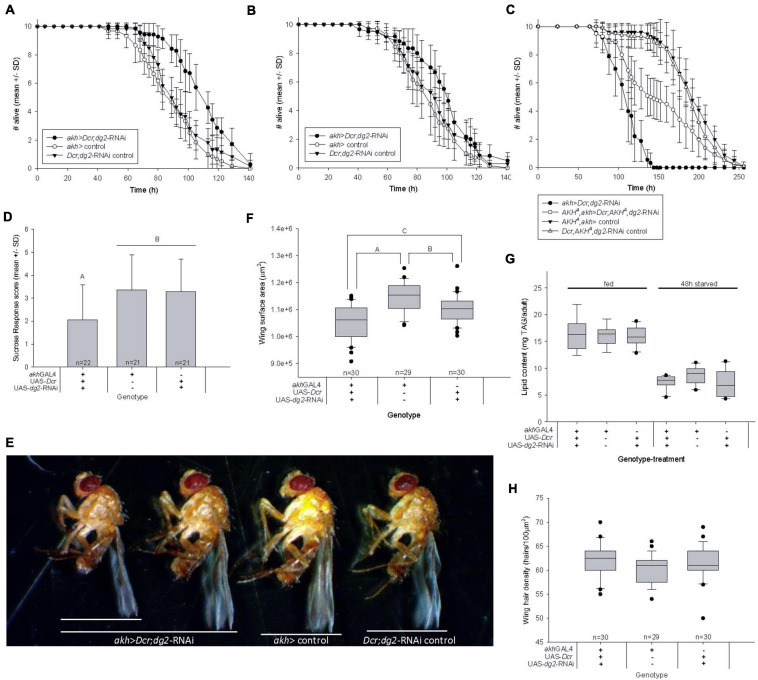
Reduced *CC-dg2* interacts with juvenile metabolic stress to alter adult metabolism and cause obesity. **(A)** Adult female starvation resistance increased significantly (*p* < 0.001) in flies reared as larvae on low nutrient food. **(B)** Adult female starvation resistance was not significantly affected (*p* = 0.146) when flies were reared as larvae on standard nutrient food. **(C)** The effect of reducing *CC-dg2* expression in a wild type AKH^+^ background on adult female starvation resistance is reversed in the AKH^A^ single mutant background. **(D)** Female sucrose response decreased significantly following 48 h starvation. Letters denote statistically significant differences (*p* = 0.011). Data are shown as mean ± standard deviation (SD). **(E)** Representative images of adult females showing body size range within *akh* > *Dcr;dg2-RNA* genotype. **(F)** Adult female body size is significantly reduced relative to controls (*n* = 40/genotype). Letters denote statistically significant differences: A *p* < 0.001; B *p* = 0.004; C *p* = 0.006 (One way ANOVA, Holm-Sidak). **(G)** Whole body TAG content per adult body was not significantly affected by reduced *CC-dg2* expression in fed (*p* = 0.966) or 48 h starved (*p* = 0.111) females. *n* = 7–9 for fed samples, *n* = 9 for 48 h starved samples. **(H)** Wing cell density is not significantly affected (*p* = 0.073). See section “Materials and Methods” for details of statistical testing.

## Results

We investigated a putative role for *dg2* in the CC (i.e., CC-*dg2*) as a regulator of AKH secretion during early development using the GAL4-UAS binary system (Brand and Perrimon, 1993). We used a CC-specific driver, *akh*-GAL4 (Lee and Park, 2004), to silence *dg2* expression specifically in the CC using a *dg2*-RNAi transgene (*dg2^*COMa*7^*-RNAi, Dason et al., 2019) in conjunction with UAS-*Dcr*. The reduction of *dg2* expression in the CC is hereafter referred to as *akh* > *Dcr*;*dg2*-RNAi. We focused this transgenic manipulation to the embryonic, larval and pupal stages by using temperature to manipulate GAL4-UAS activity (Yamamoto-Hino and Goto, 2013). GAL4 transcriptional activity is increased at high (30°C) temperatures relative to low (18°C) temperatures. We increased GAL4-UAS activity during embryonic, larval and pupal stages by rearing all flies at 30°C, and transferred newly eclosed adult flies to 18°C in order to reduce GAL4-UAS activity (Yamamoto-Hino and Goto, 2013).

From our hypothesis that *dg2* negatively regulates AKH secretion – and because AKH secretion increases in response to starvation – we predicted that the effect of *akh* > *Dcr*;*dg2*-RNAi would be apparent only under conditions of larval nutrient stress. We used a low nutrient food containing 25% of the sucrose and yeast contained in our normal nutrient fly food for larval rearing.

### CC-*dg2* and Nutrient Stress Prolonged Larval Development

*akh* > *Dcr*;*dg2*-RNAi in normal nutrient rearing conditions did not affect larval wandering (Figure 1A.I), pupariation (Figure 1A.II), or eclosion (Figure 1A.III). However, in low nutrient rearing conditions *akh* > *Dcr*;*dg2*-RNAi prolonged larval wandering (Figure 1B.I) and significantly delayed pupariation (Figure 1B.II). Larvae that showed prolonged wandering alternated between wandering and feeding before either dying in the food or as elongated prepupae on the vial walls.

Survival through larval development to pupariation was significantly reduced by *akh* > *Dcr*;*dg2*-RNAi (*p* < 0.05). In the GAL4-UAS treatment group (i.e., *akh* > *Dcr*;*dg2*-RNAi), an average (±SD) of 26.6 ± 2.0 larvae out of the 30 larvae placed in each vial at *t* = 0 h AEH survived to pupariation (Figure 1B.II). In contrast, 29.4 ± 0.7 out of 30 larvae in the *akh*-GAL4 control group and 28.9 ± 1.1 out of 30 larvae in the UAS-*dg2*-RNAi control group survived (Figure 1B.II). Proportionately, 26.6/30 (i.e., 85%) *akh* > *Dcr*;*dg2*-RNAi larvae survived vs. 29.4/30 (i.e., 98.0%) *akh*-GAL4 control larvae and 28.9/30 (i.e., 96.3%) UAS-*dg2*-RNAi control larvae.

*akh* > *Dcr*;*dg2*-RNAi affected survival through pupal metamorphosis to adult eclosion (Figure 1B.III). Out of 26.6 ± 2.0 larvae that survived to pupariation in the *akh* > *Dcr*;*dg2*-RNAi treatment group, 25.3 ± 1.5 of these survived to eclosion as adults (i.e., 25.3/26.6, or 95.2%) (Figure 1B.III). In contrast, 25.3 ± 1.5 out of 29.4 ± 0.7 larvae (86.0%) in the *akh*-GAL4 control group and 25.1 ± 2.3 out of 28.9 ± 1.1 larvae (86.5%) in the UAS-*dg2*-RNAi control group survived (Figure 1B.III). This indicated that although the total number of eclosions did not differ between the three genotype groups, *akh* > *Dcr*;*dg2*-RNAi proportionately increased survival through metamorphosis to eclosion.

These developmental effects were largely absent in response to the expression of *gfp*-RNAi in the CC, and were thus not an artifact of activating RNAi machinery (Supplementary Figure 1Ai–iii). *dg2* expression in the CC influenced larval development in a nutrient stress-dependent manner. The data reported hereafter were collected from flies reared as larvae on low nutrient food, and as adults on standard nutrient food.

### CC-*dg2* Developmental Effects Were Dependent Upon AKH

We hypothesized that the effects of *akh* > *Dcr*;*dg2*-RNAi in low nutrient rearing conditions were mediated by AKH signaling. To test this, we drove *akh* > *Dcr*;*dg2*-RNAi in *akh* genetic mutant backgrounds. Bioactive AKH is synthesized from a prepropeptide from which the APRP is also produced. Because APRP putatively regulates the developmental transitions between larval, pupal, and adult life stages in insects, we investigated the effect of *akh* > *Dcr*;*dg2*-RNAi in AKH single (AKH^A^) and AKH-APRP double (AKH^AP^) mutant backgrounds (Clynen et al., 2004; Gàlikovà et al., 2015). The statistically significant developmental delays seen with *akh* > *Dcr*;*dg2*-RNAi in a wild type AKH^+^ background were lost in both the AKH^A^ (Figure 1C.I–III) and AKH^AP^ (Supplementary Figure 1Bi–iii) mutant backgrounds. These data demonstrated that AKH mediated the developmental effects of *akh* > *Dcr*;*dg2*-RNAi in a low nutrient rearing environment.

We tested our prediction that *akh* > *Dcr*;*dg2*-RNAi increased CC secretion using *dTrpA1*. *dTrpA1* encodes a Ca^2+^-permeable cation channel that stimulates depolarization in neural and neuroendocrine tissues at 30°C, and as such provided an appropriate means of promoting secretion from the CC (Hamada et al., 2008). We drove *dTrpA1* expression at 30°C with *akh*-GAL4 (i.e., *akh* > *dTrpA1*) and phenocopied the developmental effects of *akh* > *Dcr*;*dg2*-RNAi in a low nutrient environment (Supplementary Figure 1Ci–iii). These effects were significantly reduced in larvae reared in normal nutrient conditions (Supplementary Figure 1Di–iii).

### AKH May Mediate CC-PG Paracrine Signaling

Immunohistochemistry experiments using an AKH antibody revealed the influence of *akh* > *Dcr*;*dg2*-RNAi on AKH biodynamics. The larval CC was reported to contain 119 ± 21 fmol of AKH peptide, and AKH synthesis was independent of *akh* expression (Noyes et al., 1990). Heat mapping revealed significantly more AKH-immunoreactivity (IR) in CC tissues from 92h AEH control larvae (Figures 2A,C) as compared to *akh* > *Dcr*;*dg2*-RNAi (Figures 2B,C).

*akh* > *Dcr*;*dg2*-RNAi caused AKH-dependent developmental delays in wandering and pupariation beginning at 92h AEH (Figures 1B,C). Temporally age-matched *akh* > *Dcr*;*dg2*-RNAi 92 h AEH larvae displayed developmental delay and arrest in the first and second instar, accompanied by the transparent body “glassy” phenotype (Figure 2D). Variation in developmental age was absent in 92 h AEH control larvae. This “glassy” phenotype, the developmental delays, terminal arrest and failed pupariation in third instar larvae are diagnostic of ecdysteroid dysregulation (Mirth and Riddiford, 2007). Significantly reduced CC AKH content (Figures 2A–C) coincided with the onset of developmental delay at 92 h AEH (Figures 1B.I–II, 2D). AKHergic projections from the CC that terminate on the PG were reported previously (Kim and Rulifson, 2004; Lee and Park, 2004). In light of this report, our data led us to hypothesize that AKH might influence ecdysteroid physiology through an unidentified CC-PG paracrine signaling pathway. We investigated CC axonal projections to the PG for evidence that AKH may be secreted into the PG.

We drove GFP-tagged neuronal synaptobrevin (UAS-*nSyb.eGFP*) using *akh*-GAL4 in conjunction with a UAS-*cd4:td.Tom* membrane-bound red fluorescent protein (RFP) to mark CC cell bodies and axons. GFP-tagged nSyb was reported to label synaptic vesicles and dense core granules and to mark putative sites of secretion at perisynapses (Estes et al., 2000; Zhang et al., 2002; Nässel, 2009; Shimada-Niwa and Niwa, 2014). We identified synaptobrevin clustered along RFP-labeled CC axonal projections into the PG (Figure 3AI–IV). Notably, nSyb-IR and AKH-IR colocalized in these regions. These data identified the potential for AKH secretion into the PG and suggested the existence of an unidentified CC-PG paracrine signaling pathway.

### AKH Affected Intracellular Calcium Signaling in the PG

We suspected that the putative effect of AKH signaling in the PG would be different from its catabolic activity in the fat body because the PG does not contain lipid or glycogen stores. Ecdysteroids are actively secreted from the PG via calcium-mediated vesicle exocytosis, and this signaling pathway is stimulated by an unidentified G-protein-coupled receptor (GPCR) (Yamanaka et al., 2015). The AKH receptor (AKHR) is a GPCR (Staubli et al., 2002). From these reports, we hypothesized that AKH may act through a paracrine signaling mechanism to stimulate increased cytosolic free calcium levels in the PG.

We investigated the effect of AKH signaling on PG intracellular calcium dynamics in larvae carrying either a wild type allele, *AkhR*^+^, or a loss-of-function deletion mutant allele, *AkhR*^1^, of the *AkhR* locus (Grönke et al., 2007). We conducted *in vivo* calcium imaging experiments in both *AkhR*^+^ wild type and *AkhR*^1^ mutant backgrounds using a PG-specific driver, *spok*-GAL4, to express the UAS-*GCaMP6m* calcium reporter line (Figure 4A; Chen et al., 2013; Shimell et al., 2018). Although we hypothesized that AKH acts via paracrine signaling in the PG, we were unable to inject AKH directly into the PG in order to simulate this mechanism. Instead, we incubated dissected CNS-ring glands (CNS-RG) with AKH and recorded over an hour in order to allow time for AKH to diffuse into the PG tissue. We quantified macro and microspikes of calcium activity throughout the PG, as reported previously (Yamanaka et al., 2015).

We found no significant difference between *AkhR*^+^ and *AkhR*^1^ calcium spike activity during the first two 15 min blocks of the 1h experiment (Figure 4D). Spike frequency in *AkhR*^+^ increased significantly over the course of the entire experiment; *AkhR*^+^ spike frequency was significantly greater than that of *AkhR*^1^ in the 30–45 min and 45–60 min blocks (Figure 4D). These effects are shown in representative images of *AkhR*^1^ (Figure 4BI–II) and *AkhR*^+^ (Figure 4CI–II) for 0–15 min (Figures 4BI,CI) and 45–60 min (Figures 4BII,CII) blocks.

Because AKH stimulated an increase in cytosolic free calcium levels, we quantified ecdysteroid titers in order to see if *akh* > *Dcr*;*dg2*-RNAi affected this trait. We were unable to detect changes in either circulating ecdysteroid titers (Supplementary Figure 4A) or in ecdysteroid content of dissected CNS-RG (Supplementary Figure 4B).

Regions of the CNS that project to the ring gland are themselves targets of AKH signaling (Ikeya et al., 2002; Wicher et al., 2006; Kim and Neufeld, 2015). In order to ensure that PG calcium spike activity reported in Figure 4D was a direct result of AKH signaling in the PG we used tetrodotoxin (TTX) to inhibit synaptic transmission from the CNS to the PG. In *AkhR*^+^ PG cells, the AKH + TTX treatment significantly increased the frequency of spikes relative to what was observed in response to AKH only (Supplementary Figure 4C). Notably, the number of spikes observed in the first block of the AKH + TTX experiment was not significantly different from that observed in the last block of the experiment with AKH only. These results indicated that the slow response seen in Figure 4D was due in part to antagonism of the AKH response by an unidentified CNS-derived factor. The same data for wild type with AKH only are shown in Figure 4D and Supplementary Figure 4C.

Representative videos of *AkhR*^1^ and *AkhR*^+^ (with and without TTX) responses to AKH are available online. Supplementary Videos demonstrate changes in PG intracellular calcium levels that were recorded during *in vivo* imaging experiments: *AKHR*^+^ versus *AKHR*^1^ in block 1 (Supplementary Video 1); *AKHR*^+^ versus *AKHR*^1^ in block 4 (Supplementary Video 2); and *AKHR*^+^ without TTX versus *AKHR*^+^ with TTX in block 4 (Supplementary Video 3). Note that the *AKHR*^1^ data shown in Figures 4BI,II are from the same *AKHR*^1^ tissue sample that is shown in Supplementary Videos 1 and 2; note also that the *AKHR*^+^ data shown in Figures 4CI,II are from the same *AKHR*^+^ tissue sample that is shown in Supplementary Videos 1, 2, and **3**. See Supplementary Video captions for more details.

We further demonstrated the developmental role played by AKHR in the PG by driving the expression of *AkhR*-RNAi with *Spok*-Gal4. This manipulation delayed time to pupariation (Figure 4E). Finally, we compared larval developmental timing between *AkhR*^1^, AKH^A^, AKH^AP^ mutants and the wild type control genotype and found that mutation of the *AkhR* and *akh* loci delayed wandering and significantly delayed pupariation (Supplementary Figure 4Di–ii). These data demonstrated that AKH acted through AKHR to stimulate increased intracellular calcium levels in the PG, and that dysregulated *AkhR* function caused developmental delay.

### Larval Nutrient Experience Altered Adult Metabolic Traits

Early life nutritional stress is strongly associated with adverse outcomes for adult health (Sullivan and Grove, 2010; Wang et al., 2017). We reared flies as larvae in low nutrient conditions and as adults in normal nutrient conditions, and found that *akh* > *Dcr*;*dg2*-RNAi increased adult starvation resistance significantly in virgin females (Figure 5A) and males (Supplementary Figure 5A). These results were replicated in females (Supplementary Figure 5B) and males (Supplementary Figure 5C) using another *dg2*-RNAi construct (VDRC GD38320). Consistent with the effect on larval development of *akh* > *Dcr*;*dg2*-RNAi (Figure 1A.I–III), starvation resistance in adults that were reared on normal nutrient food during larval life was not affected (Figure 5B), and was not attributable to the activation of RNAi machinery (Supplementary Figures 5A,D).

We used AKH^A^ and AKH^AP^ mutants to implicate AKH signaling in the effect of CC-*dg2* on adult starvation resistance. Whereas *akh* > *Dcr*;*dg2*-RNAi in a wild type AKH^+^ background increased adult female starvation resistance (Figure 5A), *akh* > *Dcr*;*dg2*-RNAi starvation resistance decreased significantly in the AKH^A^ single mutant background where AKH was inactive but bioactive APRP was produced (Figure 5C). The effect of *akh* > *Dcr*;*dg2*-RNAi was more severe in the AKH^+^ than in AKH^A^. There was no effect of *akh* > *Dcr*;*dg2*-RNAi in the AKH^AP^ double mutant background (Supplementary Figure 5E).

Starvation resistance influences a fly’s need to search for and ingest food, where greater resistance confers a less immediate need to feed (Dethier, 1976). The proboscis extension response (PER) assay quantifies the likelihood that a fly will ingest sucrose (i.e., SR) (Hughson et al., 2018). Starvation modulates higher-order gustatory circuits to promote PER and increase SR (Kain and Dahanukar, 2015). *akh* > *Dcr*;*dg2*-RNAi caused a significant decrease in virgin female SR following 48h starvation (Figure 5D). These data supported our finding that *akh* > *Dcr*;*dg2*-RNAi increased adult starvation resistance.

Starvation resistance correlates with whole body lipid content, and whole body lipid content is typically directly proportional to body size (Chippindale et al., 1996; Vermeulen et al., 2006). Adult wing surface area is directly proportional to body size, and *akh* > *Dcr*;*dg2*-RNAi significantly decreased female body size (Figures 5E,F). In *D. melanogaster* literature, the assumption that body size is always directly proportional to whole body protein content underlies the practice of standardizing TAG data to protein content. In contrast to the significant reduction in adult wing surface area (Figure 5F), we found that *akh* > *Dcr*;*dg2*-RNAi did not affect whole body protein content in fed flies (Supplementary Figure 5F). Furthermore, 48 h starvation significantly altered whole body protein content between genotypes (Supplementary Figure 5F). Whole body protein content was neither an appropriate surrogate for body size in our experiments, nor was whole body protein content stable across feeding treatments. We report whole body TAG content per individual.

Human obesity was clinically defined as a disproportionately high body mass relative to body height, where height is used as a surrogate measure of body size (Gutin, 2018). Obesity can be characterized in flies by examining whole body TAG content in proportion to body size. TAG content per individual fly was the same across genotypes (Figure 5G), and this indicated that – in proportion to body size (Figure 5F) – there was more TAG in the small-bodied adults that experienced *akh* > *Dcr*;*dg2*-RNAi and nutrient stress during larval life. These data suggested that adult obesity was a developmental outcome of this life stage-specific transgenic and nutritional manipulation. This may underlie the reported increase in adult starvation resistance.

Adult body size is determined by both cell proliferation and cell growth/size during larval life, and ecdysteroids influence these processes. Wing cells each produce one hair, and differences in wing hair density thus reflect differences in cell size. *akh* > *Dcr*;*dg2*-RNAi did not affect wing hair density in a 100 μm^2^ area (Figure 5H). The small body size of *akh* > *Dcr*;*dg2*-RNAi adults was not a consequence of reduced cell growth during larval development.

## Discussion

We identified a nutrient-dependent developmental role for AKH; this is the first evidence of its kind in *D. melanogaster*. Downregulation of CC-*dg2* expression in low nutrient rearing environments increased larval lethality and delayed the developmental transition from larval to pupal life. Flies that survived this treatment were smaller and more resistant to nutrient stress during adulthood. We discovered that AKH increased PG cytosolic free calcium levels, and this supported previous work that identified the potential for CC-PG paracrine signaling (Kim and Rulifson, 2004; Lee and Park, 2004; Yamanaka et al., 2015). Here we discuss the major findings and implications of our research: (1) *dg2*/PKG as a regulator of AKH activity and nutrient stress responsivity, (2) a novel developmental role for AKH, (3) the effect of AKH on PG intracellular calcium levels, and (4) a model for AKH as a nutrient-responsive prothoracicotropic factor, and its relevance to human development and metabolism.

### *dg2* Regulated AKH Nutrient Stress Responsivity

In starving flies, AKH is secreted from the CC and acts on various peripheral targets. AKH stimulates catabolism of glycogen and lipid reserves for an immediate source of energy (Kim and Rulifson, 2004; Isabel et al., 2005;(Lee and Park, 2004; Grönke et al., 2007; Stoffolano et al., 2014; Gàlikovà et al., 2015), stimulates locomotion in order to promote foraging behavior (Yu et al., 2016), increases sweet gustatory receptor neuron excitability so that starving flies will ingest low quality food (Dethier, 1976; Jourjine et al., 2016), and regulates post-prandial insulin secretion (Kim and Neufeld, 2015). The effect of metabolic stress on aging and longevity is also mediated by AKH (Waterson et al., 2014). These diverse functions implicate AKH in orchestrating a suite of changes in physiology and behavior that promote survival and restore metabolic homeostasis. Our work contributed this model: AKH signaling modified larval development in response to low nutrient stress, and *dg2* expression in the CC regulated this effect.

### *dg2* Altered AKH Activity

*dg2* expression in the CC interacted with AKH to mediate the developmental response to low nutrient stress. Although we did not report definitive evidence of altered AKH secretion in response to *akh* > *Dcr*;*dg2*-RNAi, this is consistent with the *D. melanogaster* literature. Previous reports assayed the same phenotypes that we did for providing surrogate evidence of changes in AKH secretion. These included changes in the CC intracellular AKH content (Braco et al., 2012; Kim and Neufeld, 2015), altered lifespan during starvation (Braco et al., 2012; Gàlikovà et al., 2015), changes in metabolism and behavior (Lee and Park, 2004; Isabel et al., 2005), and *in vivo* imaging assays (Kim and Rulifson, 2004; Jourjine et al., 2016). Altogether, our data supported a role for *dg2* as a negative regulator of AKH secretion.

*akh* > *dTrpA1* stimulated secretion from the CC and phenocopied the developmental effect of *akh* > *Dcr*;*dg2*-RNAi. Increased activity of Ca^2+^ channels promotes AKH secretion from the CC, and the DTRPA1 Ca^2+^-permeable cation channel provides a strong secretory stimulus (Pannabecker and Orchard, 1989; Hamada et al., 2008). The stronger effect of *akh* > *dTrpA1* might be attributable to the ability of its strong secretory stimulus to override other regulatory inputs that mitigated the effect of *dg2*-RNAi (Braco et al., 2012).

Future investigations into the means whereby *dg2* regulates AKH activity will focus on the influence of PKG activity on CC intracellular Ca^2+^. AMPK and cAMP signaling promote CC cell excitability and increases AKH secretion by mediating an increase in intracellular Ca^2+^ in both *D. melanogaster* and *Locusta migratoria* (Pannabecker and Orchard, 1989; Braco et al., 2012). AMPK and cAMP signaling likewise promote intracellular Ca^2+^ and glucagon secretion from murine α cells, and PKG activity inhibits glucagon secretion by preventing Ca^2+^-mediated membrane depolarization (Leiss et al., 2011). This promising avenue of research is supported by the data we report herein.

New methods of haemolymph extraction permit precise sampling required for hormone titer analysis (MacMillan and Hughson, 2014). The establishment of *D. melanogaster* as a model organism in metabolic syndrome research now requires the development of bioassays sensitive enough to quantify biologically significant changes in AKH titers.

### AKH Altered Larval Development

Larval development in low nutrient rearing conditions was delayed by 6 h in response to *akh* > *Dcr*;*dg2*-RNAi in an AKH^+^ genetic background. Delays of only 3 h exert significant effects on growth and survival (Bakker, 1961). The developmental delay caused by *akh* > *Dcr*;*dg2*-RNAi in the AKH^+^ background was lost in both the AKH^A^ single mutant (where bioactive APRP is produced) and AKH^AP^ double mutant backgrounds (where neither AKH nor APRP are bioactive). From these data, *dg2* acted in the CC to regulate AKH signaling and this mediated a developmental response to low nutrient stress. This fits with the established role of the CC as a stress-responsive tissue (Vogt, 1946). Consistent with our findings, previous studies reported that AKH was dispensable during larval development in nutrient-abundant conditions (Kim and Rulifson, 2004; Lee and Park, 2004; Isabel et al., 2005; Grönke et al., 2007; Bharucha et al., 2008; Gàlikovà et al., 2015).

Insulin signaling affects larval development by acting directly on the PG to alter ecdysteroid biosynthesis (Caldwell et al., 2005; Colombani et al., 2005; Mirth et al., 2005). AKH altered insulin signaling through protein-dependent secretion of DILP3 (Kim and Neufeld, 2015). Yeast – the protein source in our low nutrient diet – provides cholesterol for ecdysteroid synthesis. From this, the possibility that *akh* > *Dcr*;*dg2*-RNAi and low nutrient diet influenced development through AKH signaling to the IPCs must be further investigated; this is discussed in section 4.3.2.

Adipokinetic hormone precursor-related peptide was hypothesized to regulate developmental transitions (Clynen et al., 2004; De Loof et al., 2009). Seminal work on the AKH^A^ and AKH^AP^ mutants reported increased starvation resistance in these mutant lines relative to controls (Gàlikovà et al., 2015). Our data unexpectedly demonstrated that adult starvation resistance was significantly reduced in *akh* > *Dcr*;*dg2*-RNAi flies when AKH was inactive but APRP was active (i.e., AKH^A^; Figure 5C), and there was no effect when both AKH and APRP were inactive (i.e., AKH^AP^, Supplementary Figure 5E). The work reported by Gàlikovà et al. (2015) used flies reared as larvae in a nutrient-abundant environment and APRP did not alter adult starvation resistance. In light of this seminal report, we emphasize that our data must not be viewed as evidence of a direct role for APRP but rather of the complexity of tissue-specific gene function during early life development and its responsivity to a stressful nutrient environment. We propose that a putative role for APRP during pupal metamorphosis be investigated.

### AKH Altered PG Calcium Dynamics

Adipokinetic hormone increased cytosolic free calcium levels in the PG. We hypothesized that AKHR might be an unidentified GPCR that stimulated the signaling cascade for vesicle-mediated ecdysteroid secretion from the PG (Yamanaka et al., 2015). While AKHR might not be present in the PG and it is possible that AKH acts indirectly on the PG, our neuroanatomical data and TTX experiments suggested that AKH might act directly on the PG. Here we propose a model wherein AKH acted directly on the PG to alter development in response to low nutrient stress.

AKHR mediated a stimulatory effect of AKH on PG calcium dynamics, but the response to AKH was slower than expected for GPCR signaling. We are not first to report slow intracellular calcium responsivity to stimulated GPCR signaling. In *Rhodnius*, malphighian tubules incubated in a serotonin solution exhibited changes in intracellular calcium levels that occurred over the order of 100–200 s (Gioino et al., 2014).

This slow response can be explained by our method for AKH treatment in this assay. Our model proposed a paracrine mechanism through which AKH was secreted directly into the PG. In contrast, we incubated dissected CNS-RG tissues in an AKH solution; this required the passive diffusion of AKH into the PG. It is thus possible that delayed and/or reduced AKH delivery to target cells in the PG caused the slow response. AKH injection into pupae was reported to increase heart rate slowly and only at concentrations of AKH two to five times greater than what is contained in the CC (Noyes et al., 1990). This was explained by the inability of AKH injection to replicate the high potency of endogenous paracrine secretion of AKH from the CC directly onto the aorta. Additionally, the long duration of our assay required that the CNS-RG be removed from the cuticle. As with Yamanaka et al. (2015), we found that this reduced spontaneous calcium spike frequency in the PG; cuticle removal may similarly have reduced PG sensitivity to AKH. Finally, it is possible that AKH acts indirectly on the PG by stimulating chemical-mediated exocytosis of a secreted factor that then acts on the PG to affect intracellular calcium dynamics.

### Other CNS-RG-Derived Factors Influenced PG Calcium Dynamics

Another possibility for the slow calcium response to AKH might be that AKH signaling acts indirectly to make the gland competent to receive another GPCR signal, or that the response is modulated by other signals. Consistent with this view is our observation that application of TTX with AKH in our calcium imaging experiments significantly increased spike activity in *AkhR*^+^ PGs relative to the application of AKH alone. This suggests that the CNS produces a signal that antagonizes the stimulatory effect of AKH in the PG.

Multiple prothoracicotropic and prothoracicostatic factors target the PG in order to regulate ecdysteroid physiology, and their interactions during development are not well characterized. Hormones like PTTH, insulin and serotonin have tropic effects on ecdysteroidogenesis in insects, while prothoracicostatic peptide (PTSP) inhibits this process (Colombani et al., 2005; McBrayer et al., 2007; Yamanaka et al., 2010; Shimada-Niwa and Niwa, 2014). PTTH, insulin, and serotonin communicate nutritional information to the PG in order to modify development in response to the environment. AKH may interact with one or more prothoracicotropic and/or –static factors in order to coordinate ecdysteroid biosynthesis and secretion. Whether or not AKH indeed interacts with another factor, and whether or not this interaction directly regulates ecdysteroid secretion or indirectly permits PG competency, are subjects for future investigations.

### Is AKH a Novel Prothoracicotropic Factor?

The PG is a decision-making center that regulates ecdysteroid physiology so as to modify growth and development in response to nutritional inputs (Yamanaka et al., 2013). IIS and PTTH provide nutritional information to the PG pertaining to the anabolic status of the larva (Caldwell et al., 2005; Colombani et al., 2005; Mirth et al., 2005). We hypothesize that AKH is a nutrient stress-responsive factor that communicates catabolic status to the PG. The precise effect of AKH on ecdysteroid physiology must be investigated in future work.

When essential nutrients are deficient, starving larvae must make the decision to pupariate. Starvation inhibits anabolism, and larvae cannot mount an adaptive response through IIS/TOR (Layalle et al., 2008). Starvation promotes catabolism, which stimulates AKH secretion. We showed that AKH signaling in the PG stimulated cytosolic free calcium levels in a manner reminiscent of that reported for the active secretion of ecdysteroids. We propose that *dg2*/PKG-mediated AKH secretion from the CC stimulates ecdysteroid secretion in response to low nutrient stress. Possible evidence for this model lies in the reduction in wing cell proliferation caused by *akh* > *Dcr*;*dg2*-RNAi (Figure 5H), as this effect was associated with a modest increase in haemolymph ecdysteroid titers throughout larval life (Colombani et al., 2005).

We were unable to detect a change in circulating ecdysteroid titers in response to *akh* > *Dcr*;*dg2*-RNAi, and there are several possible explanations for this. First, quantification of both ecdysone and 20-hydroxyecdysone may be required to detect changes in ecdysteroid secretion, as in Yamanaka et al. (2015). Second, more frequent sampling of ecdysteroid titers during the third larval instar might increase the resolution of ecdysteroid titer analysis. Third, calcium imaging experiments with TTX identified a putative prothoracicostatic factor(s) that targeted the PG and attenuated the effect of AKH; the effects of competing inputs were attributed to slower cardioacceleratory response to AKH (Noyes et al., 1990). In the future, the influence of multiple signaling inputs to the PG must be controlled for in the characterization of ecdysteroid secretory dynamics.

Loss of *AkhR* in the PG caused developmental delay (Figure 4E), but AKH signaling also caused developmental delay (Figures 1B,C, 4E). This may be explained by functional redundancy in the regulatory input of nutritional information to the PG. Decreased serotonin signaling in the PG delayed development in response to low nutrient stress (Shimada-Niwa and Niwa, 2014). Serotonin can cause developmental delay in response to low nutrient stress independently of AKH signaling in the PG. Chronically dysregulated systemic AKH signaling in *akh* > *Dcr*;*dg2*-RNAi larvae might also contribute to the delay.

In a model where AKH signaling in the PG stimulated ecdysteroid secretion, why were larvae developmentally delayed in response to *akh* > *Dcr*;*dg2*-RNAi We suggest that *akh* > *Dcr*;*dg2*-RNAi stimulated ecdysteroid secretion throughout larval life and depleted PG ecdysteroid content. Support for this comes from Yamanaka and colleagues, who observed first and second instar larval lethality in response to activated GPCR signaling in the PG: precise temporal regulation of GPCR signaling is essential for developmental progression through larval life (Yamanaka et al., 2015).

### AKH and Metabolic Syndrome

Chronic dysregulation of glucagon and insulin signaling underlie the pathogenesis of type 2 diabetes mellitus T2DM, and the activities of both hormones must be studied together (Unger and Orci, 1975). DILPs have been the primary focus of metabolic disease modeling in *D. melanogaster* (Owusu-Ansah and Perrimon, 2014). Our work demonstrates that the CC-specific role of PKG as a regulator of AKH activity must be considered in the development of fly models for metabolic syndrome.

In humans, pre-natal, childhood, and pubertal malnutrition perturb GnRH signaling in the HPG axis, and thereby contribute to the pathogenesis of metabolic syndrome both within and across generations (Habtu et al., 1999; Painter et al., 2008; Jang et al., 2013; Wang et al., 2017). We reported that CC-*dg2*-mediated disruption of AKH signaling during embryonic, larval, and pupal development – in conjunction with larval nutrient stress – increased the whole body lipid to body size ratio in a manner suggestive of obesity: this suggests a functional parallel between AKH and GnRH. The GnRHR ortholog, *AkhR*, has been implicated in the intergenerational transmission of nutrient stress effects on lipid homeostasis (Palu et al., 2017). Glucagon and GnRH are evolutionarily related (Lindemans et al., 2009; Zandawala et al., 2017). The dual functionality of AKH as a glucagon-like and a GnRH-like peptide presents great potential for understanding the etiological basis of metabolic syndrome, as well as the means whereby the effects of nutrient stress are transmitted across generations through altered HPG axis activity.

## Data Availability Statement

The raw data supporting the conclusions of this article will be made available by the authors, without undue reservation.

## Author Contributions

MS and MBO carried out the developmental timing assays in Figure 4E and ecdysteroid titer quantification in Supplementary Figures 4A,B. BNH performed the other experiments. All authors contributed to the article and approved the submitted version.

## Conflict of Interest

The authors declare that the research was conducted in the absence of any commercial or financial relationships that could be construed as a potential conflict of interest.

## Footnotes

1 https://fiji.sc/

## Supplementary Material

Supplementary Video 1 wild type *A**K**H**R*^+^ (left panel) and *A**K**H**R*^1^ mutant (right panel). Video shows changes in PG intracellular calcium levels during block 1 (0 to 15 minutes). AKH was added at t = 5 minutes. The warmer colours indicate higher levels of calcium.

Supplementary Video 2 wild type *A**K**H**R*^+^ (left panel) and *A**K**H**R*^1^ mutant (right panel). Video shows changes in PG intracellular calcium levels during block 4 (45 to 60 minutes). The warmer colours indicate higher levels of calcium. Note that these block 4 *A**K**H**R*^+^ and *A**K**H**R*^1^ samples are the same tissues as shown for block 1 *A**K**H**R*^+^ and *A**K**H**R*^1^ samples in Supplementary Video 1.

Supplementary Video 3 wild type *A**K**H**R*^+^ without TTX (left panel) and wild type *A**K**H**R*^+^ with TTX (right panel). Video shows changes in PG intracellular calcium levels during block 4 (45 to 60 minutes). The warmer colours indicate higher levels of calcium. Note that the *A**K**H**R*^+^ without TTX block 4 sample shown here in the left panel is the same dataset as shown for the block 4 *A**K**H**R*^+^ sample in Supplementary Video 2.
