# Supplementary figures and images for: AKH Signaling in D. melanogaster Alters Larval Development in a Nutrient-Dependent Manner That Influences Adult Metabolism
Source: Front Physiol. 2021 Feb 23;12:619219. doi: 10.3389/fphys.2021.619219 (PMC7940354; doi:10.3389/fphys.2021.619219)

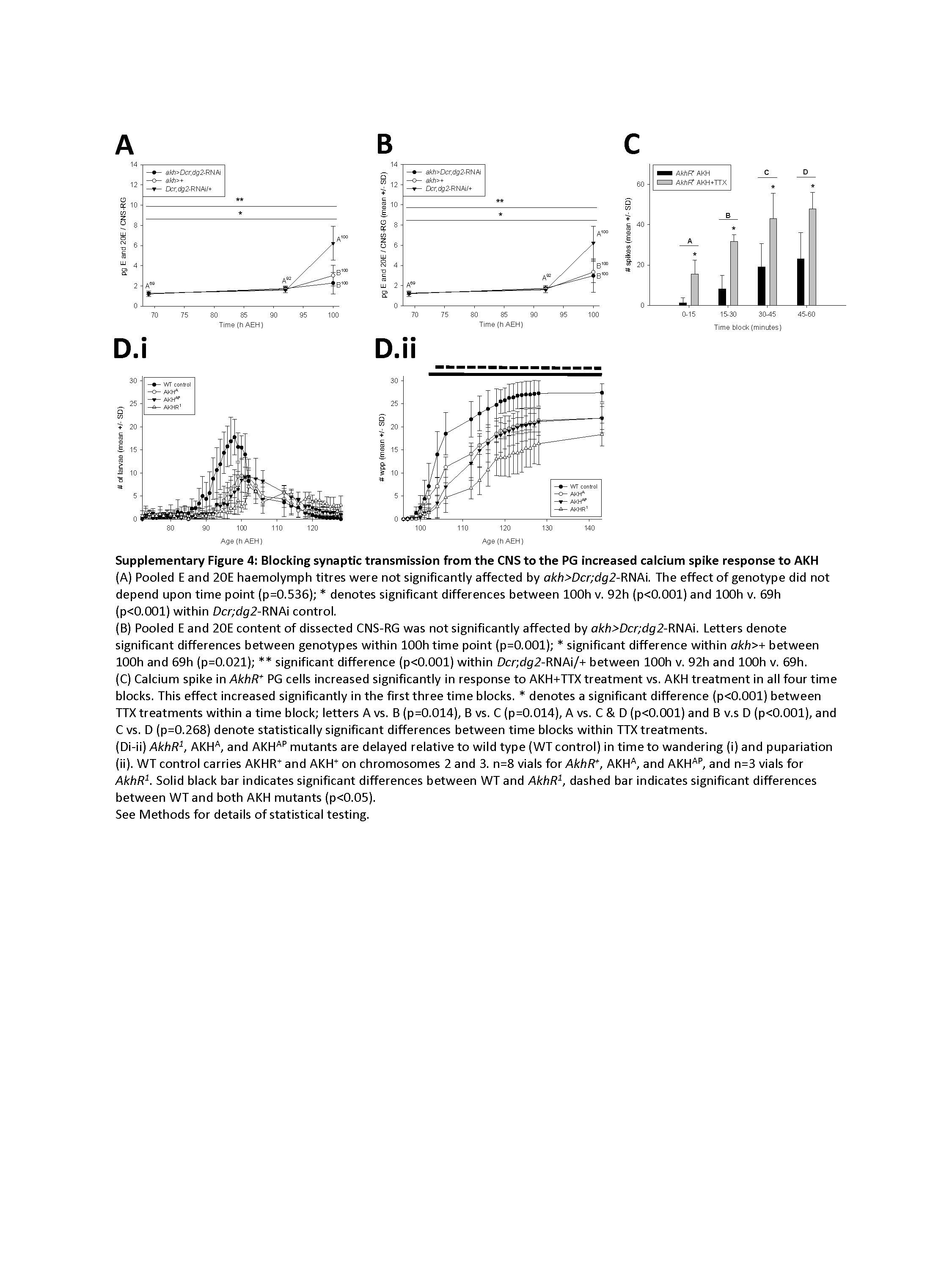

Supplement: Supplementary file 4 [file Figure_1.JPEG]

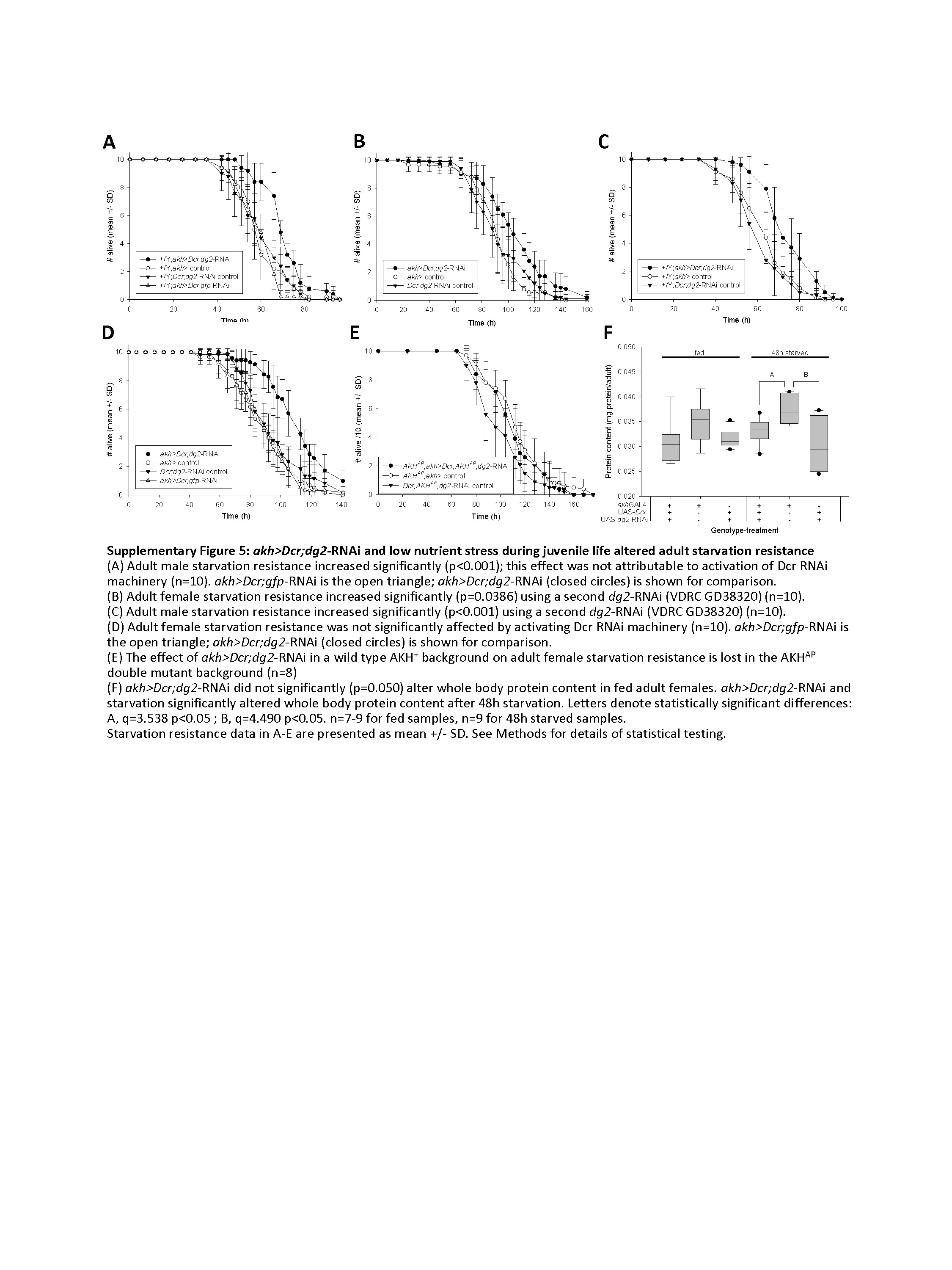

Supplement: Supplementary file 5 [file Figure_2.JPEG]

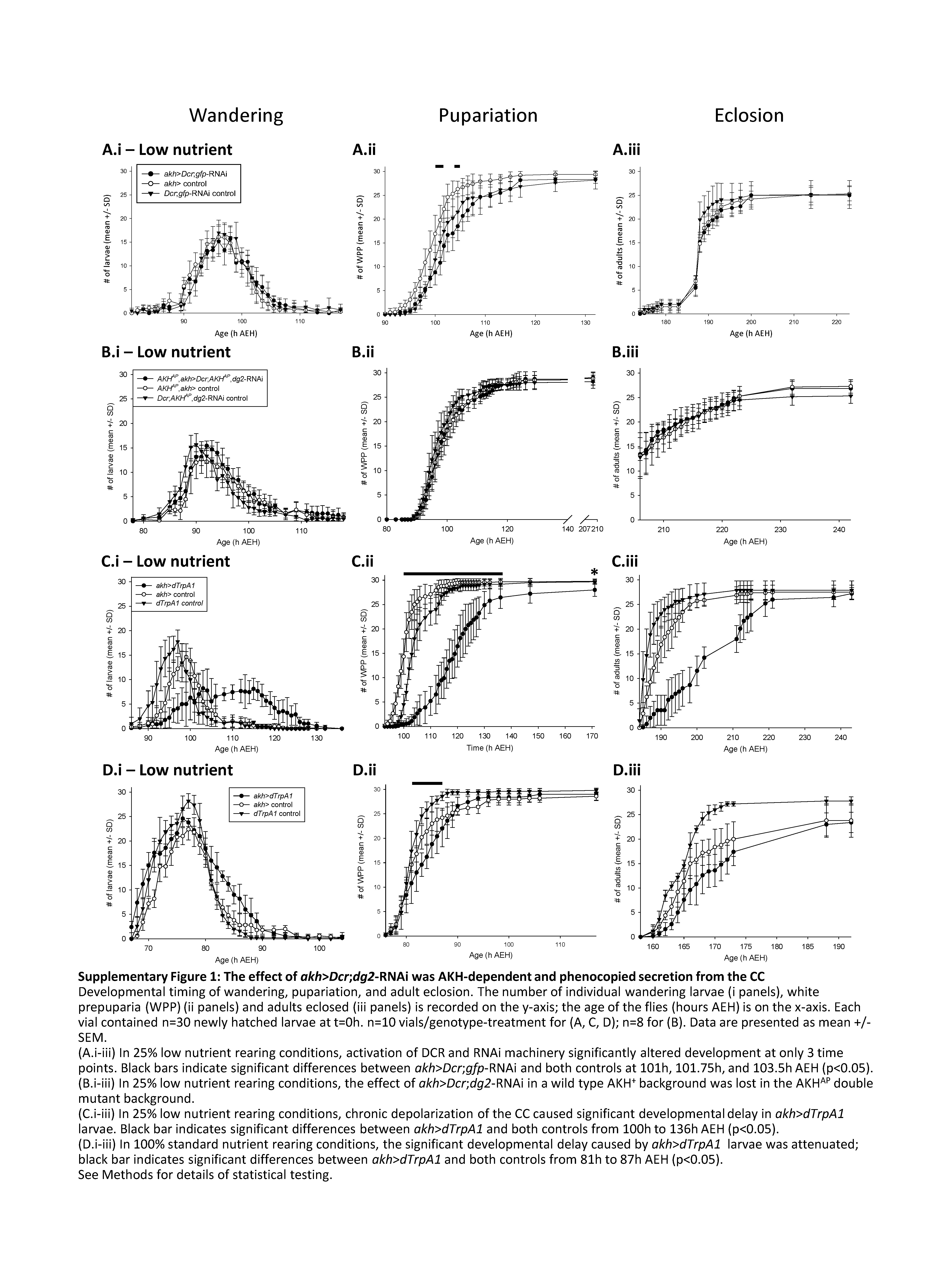

Supplement: Supplementary file 6 [file Figure_3.JPEG]
